# CT-based radiomics for predicting pathological grade in hepatocellular carcinoma

**DOI:** 10.3389/fonc.2024.1295575

**Published:** 2024-04-16

**Authors:** Yue Huang, Lingfeng Chen, Qingzhu Ding, Han Zhang, Yun Zhong, Xiang Zhang, Shangeng Weng

**Affiliations:** ^1^ Department of Hepatobiliary Pancreatic Surgery, The First Affiliated Hospital of Fujian Medical University, Fuzhou, China; ^2^ Fujian Abdominal Surgery Research Institute, The First Affiliated Hospital of Fujian Medical University, Fuzhou, China

**Keywords:** hepatocellular carcinoma, inflammatory biomarkers, pathological grade, radiomics, CT

## Abstract

**Objective:**

To construct and validate radiomics models for hepatocellular carcinoma (HCC) grade predictions based on contrast-enhanced CT (CECT).

**Methods:**

Patients with pathologically confirmed HCC after surgery and underwent CECT at our institution between January 2016 and December 2020 were enrolled and randomly divided into training and validation datasets. With tumor segmentation and feature extraction, radiomic models were constructed using univariate analysis, followed by least absolute shrinkage and selection operator (LASSO) regression. In addition, combined models with clinical factors and radiomics scores (Radscore) were constructed using logistic regression. Finally, all models were evaluated using the receiver operating characteristic (ROC) curve with the area under the curve (AUC), calibration curve, and decision curve analysis (DCA).

**Results:**

In total 242 patients were enrolled in this study, of whom 170 and 72 formed the training and validation datasets, respectively. The arterial phase and portal venous phase (AP+VP) radiomics model were evaluated as the best for predicting HCC pathological grade among all the models built in our study (AUC = 0.981 in the training dataset; AUC = 0.842 in the validation dataset) and was used to build a nomogram. Furthermore, the calibration curve and DCA indicated that the AP+VP radiomics model had a satisfactory prediction efficiency.

**Conclusions:**

Low- and high-grade HCC can be distinguished with good diagnostic performance using a CECT-based radiomics model.

## 1 Introduction

Hepatocellular carcinoma (HCC) is the most common type of primary hepatic malignant tumor (1) and the third leading cause of cancer-related deaths worldwide (2). HCC is a very heterogeneous tumor that has reduced the efficacy of clinical treatments (3, 4). The pathological grade is associated with tumor heterogeneity and the selection of different therapeutic schedules. For example, the Edmondson-Steiner grade is an important prognostic factor for curative resection of HCCs (5), while poor differentiation is a risk factor for tumor seeding or intrahepatic dissemination after radiofrequency ablation(RFA) for HCC (6). In addition, previous studies have found that one of the most important factors influencing intrahepatic recurrence is pathological grading (7). Thus, to optimize treatment and evaluate prognosis, accurate pathological grading of HCC is crucial. However, pathological grades are difficult to predict preoperatively based on clinical and serological indicators. Therefore, a noninvasive method for predicting the pathological grades of HCC is urgently needed.

Radiomics has been commonly used in clinical studies, for the conversion of medical images into high-dimensional features that can be mined quantitatively (8), and for the determination of the heterogeneity of the tumor (9, 10). Few radiomic analyses have aimed to identify the pathological grade of poorly differentiated HCCs (11–13). However, these studies have several shortcomings such as the lack of validation cohorts, the use of subjective features to build models, and the lack of clinical models for comparison. Additionally, based on previous findings, there were differences in the CT enhancement patterns of HCC depending on cellular differentiation (14, 15). Therefore, we surmised that enhanced CT images might have a more positive role than ordinary CT scan images in predicting the level of HCC tumor differentiation.

Recent studies have shown that tumor characteristics and serological indicators affect cancer progression and prognosis (16). Clinical indicators such as tumor size, derived neutrophil-to-lymphocyte ratio (dNLR), and lymphocyte-to-monocyte ratio (LMR) have been used to predict the HCC differentiation grade, but their efficiency has been unsatisfactory (17, 18). Therefore, we attempted to use CT radiomics features and inflammatory biomarkers (e.g., platelets, neutrophils and lymphocytes) to construct a combined model and evaluate its efficiency.

We designed this study to construct and validate radiomics models for HCC-grade prediction based on contrast-enhanced CT (CECT). We also compared the radiomics models and radiomics-clinical combined models using validation data to explore the best plan for prediction.

## 2 Materials and methods

### 2.1 Patients

**Figure 1:**
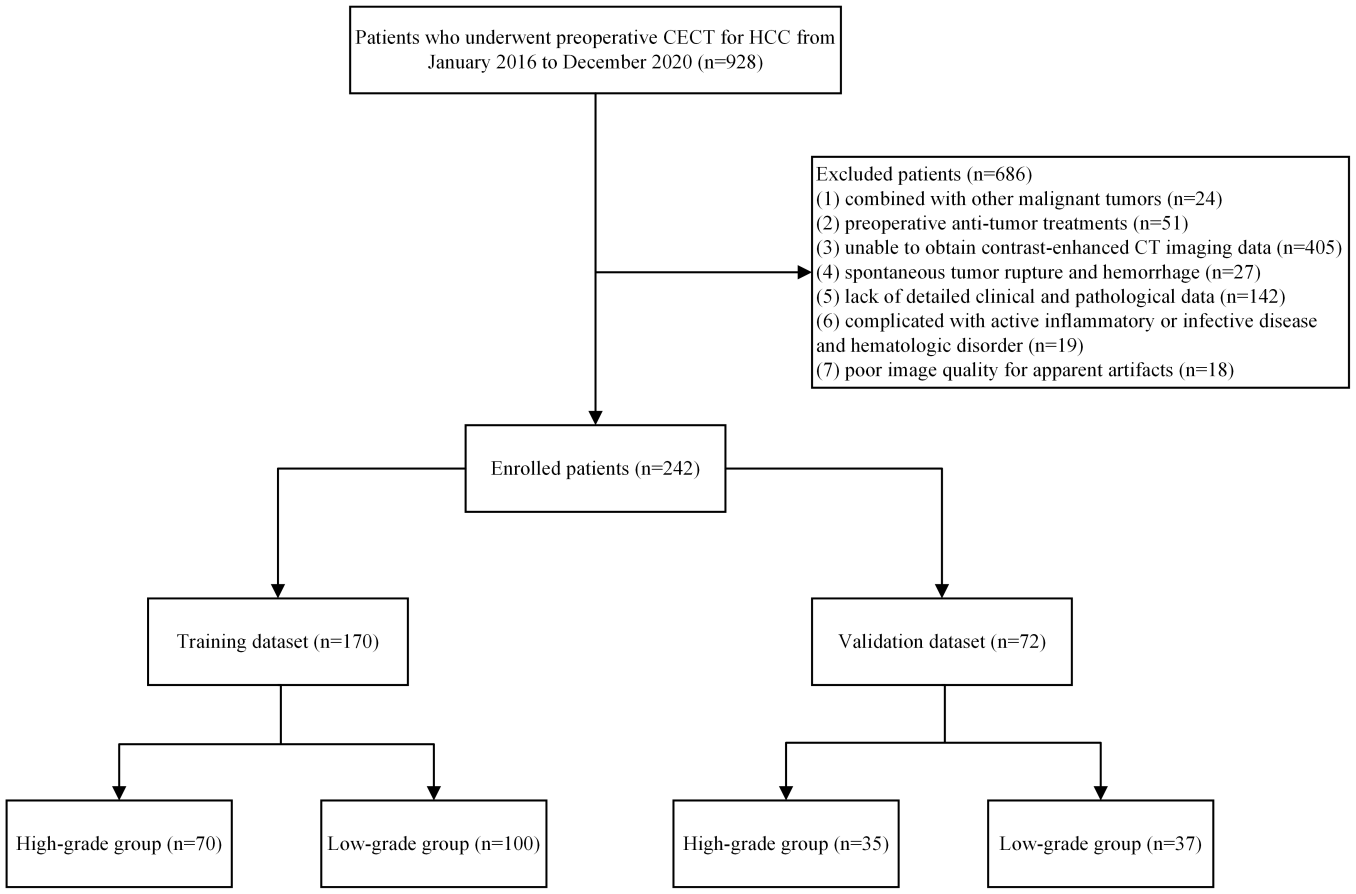
Flow chart of enrolled patients.

Following the Declaration of Helsinki, the study protocol was approved by the Ethics Committee of the First Affiliated Hospital of Fujian Medical University. This study enrolled patients that are pathologically diagnosed with HCC after surgery at the First Affiliated Hospital of Fujian Medical University between January 2016 and December 2020. The inclusion criteria were as follows: (1) pathological diagnosis with HCC after the first partial hepatectomy and (2) liver CECT no more than 1 month before surgery. The exclusion criteria were: (1) combined with other malignant tumors; (2) preoperative anti-tumor treatments; (3) enhanced CT in other hospitals and could not obtain DICOM image data; (4) spontaneous tumor rupture and hemorrhage; (5) lack of detailed clinical and pathological data; (6) complicated with active inflammatory or infective disease and hematologic disorder; (7) poor image quality for apparent artifacts. A flowchart of the patient screening is shown in **Figure 1**.

### 2.2 Clinicopathological characteristics

Preoperative clinical data were collected from the picture archiving and communication system (PACS), including demographic characteristics (age and gender) and laboratory tests including hepatitis B surface antigen (HBsAg), alpha-fetoprotein (AFP), alanine aminotransferase (ALT), aspartate aminotransferase (AST), total bilirubin (TBIL), direct bilirubin (DBIL), indirect bilirubin (IBIL), blood urea nitrogen (BUN), serum creatinine (Scr), total cholesterol (TC), triglyceride (TG), red blood cell count (RBC), neutrophil count (NEU), lymphocyte count (LYMPH), and platelet count (PLT). The neutrophil-to-lymphocyte ratio (NLR), platelet-lymphocyte ratio (PLR), and systemic immune inflammation index (SII) were calculated as follows:


NLR=neutrophil count/lymphocyte count,

PLR=platelet count/lymphocyte count,

SII = platelet count × neutrophil count/lymphocyte count

(17, 19).

Pathological data were retrieved from the Pathology Information Management System, and pathological grades were recorded according to the Edmondson-Steiner grading system. The patients were divided into low-grade (Edmondson-Steiner grade I or II) and high-grade (Edmondson-Steiner grade III or IV) groups (20). A patient may have more than one tumor, and each tumor may have a different degree of pathological differentiation; therefore, the pathological grade mentioned in the study was determined by the largest tumor (21).

### 2.3 Image acquisitions

All examinations were performed using a Toshiba Aquilion One 320-slice spiral CT or a Toshiba Aquilion Prime 80-slice spiral CT. Three-phase enhanced images were obtained for all patients. The CT parameters were as follows: tube voltage, 120 kV; tube current, 230 mAs; rotation time, 0.35 s; slice interval, 0 mm; and slice thickness, 5 mm. The nonionic contrast agent indophenol (370 mg/mL) was injected through the elbow vein at a dose of 1.5 mL/kg and a flow rate of 3.0 ml/s. Arterial phase (AP) and portal venous phase (VP) images were acquired 30 and 60 s after injection, respectively.

### 2.4 Image processing and feature extraction

**Figure 2:**
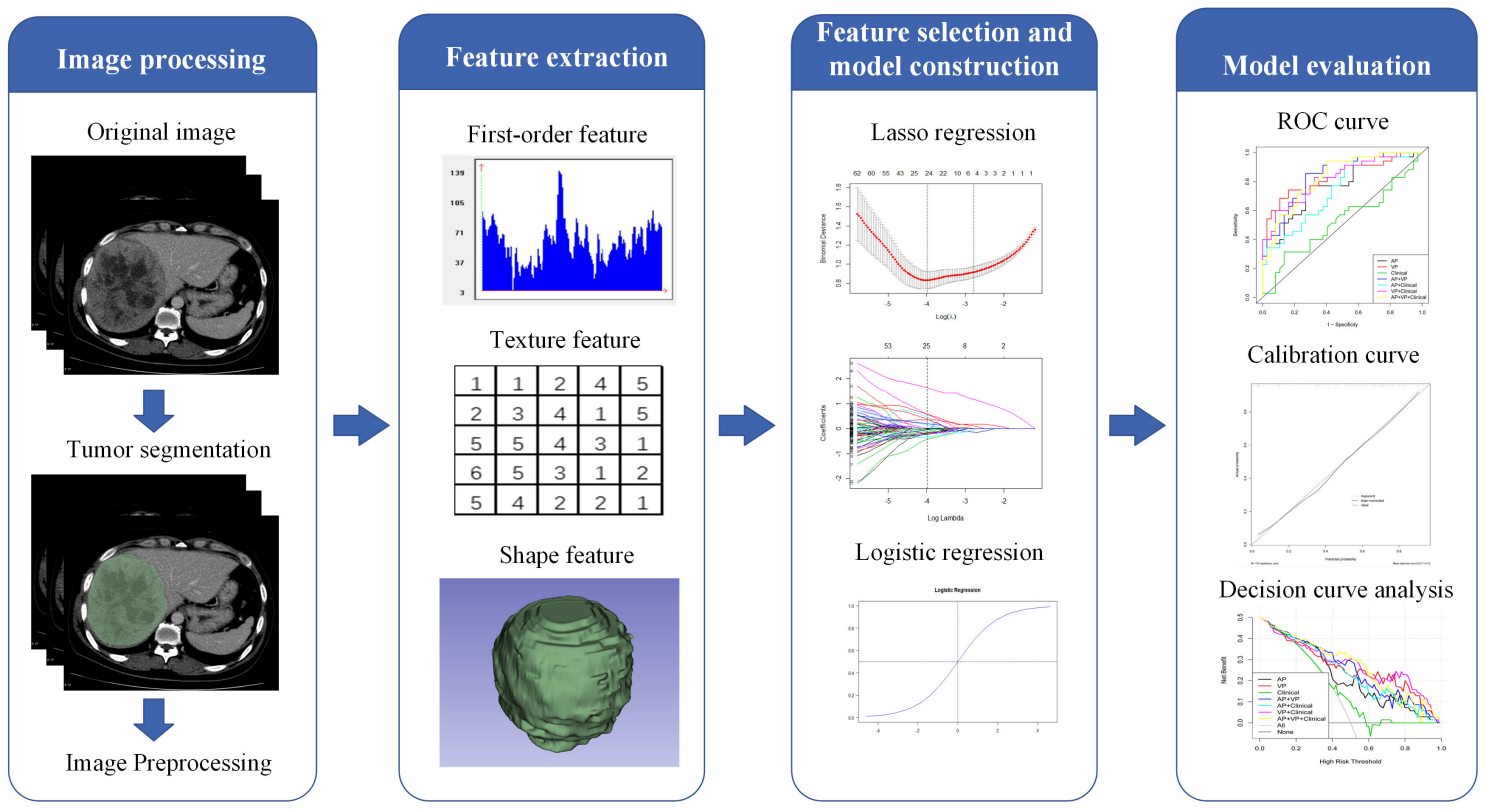
The workflow of building and validating models.

**Figure 2** illustrates the radiomics workflow. The PACS was used to retrieve the AP and VP CECT images. Using a 3D Slicer (version 4.9.0; http://www.slicer.org), reader 1 manually delineated all regions of interest (ROIs) on each transverse section of the AP and VP images. Reader 2 segmented the ROIs for 50 CT images at random. Two weeks later, reader 1 performed segmentation again to assess reproducibility. A patient may have had more than one tumor, but each ROI was only delineated from the largest tumor.

After tumor segmentation using the Pyradiomics package (version 3.0.1, https://pyradiomics.readthedocs.io/en/3.0.1/), radiomic features were extracted from both AP and VP images (normalized was true, resampled voxel size was 1 × 1 × 1 mm^3^, the gray level was discretized by a fixed bin width of 25, and the sigma values of image reconstruction were 1.0, 2.0, 3.0, 4.0, and 5.0). Radiomic features were extracted from original and filtered images, including first-order, shape, gray-level dependence matrix (GLDM), gray-level co-occurrence matrix (GLCM), gray-level run length matrix (GLRLM), gray-level size zone matrix (GLSZM), neighboring gray-tone difference matrix (NGTDM), wavelet features, and Laplacian of Gaussian filtered (LoG) features. Utilizing inter- and intra-class correlation coefficients (ICCs) calculated from reader 1 and reader 2 segmentation data, we evaluated the inter-observer reliability and reproducibility of feature extraction (22). Only features with an ICC > 0.75 were included in further analysis.

### 2.5 Radiomics feature selection and Radscore calculation

Patients were divided into the training and validation datasets at a 7:3 ratio. The training dataset was used for model construction, whereas the validation dataset was used for the performance evaluation of the constructed models.

Three steps were required to reduce the features selected from the ROIs and complete the radiomics model construction. First, only features with inter- and intra-observer ICCs greater than 0.75 were retained for the following analysis. Second, after univariate analysis, many irrelevant features (P-value > 0.05) were excluded. Third, following 10-fold cross-validation, we used the least absolute shrinkage and selection operator (LASSO) regularization to verify the penalty coefficient. LASSO was also used to predict HCC pathological grades with robust and non-redundant features alongside their corresponding coefficients before surgery. Using the selected features and their coefficients, we calculated three personalized radiomics scores (Radscores) for each patient: AP-Radscore, VP-Radscore, and AP+VP-Radscore.

### 2.6 Model construction and evaluation

Demographic characteristics and laboratory data in the low- and high-grade groups were compared using univariate analysis. Only clinical factors with a P-value< 0.05 were selected for clinical model building by multiple-factor (logistic regression) analysis. Furthermore, clinical factors from the clinical model were combined with Radscores from the radiomics model to develop a combined model and nomogram.

The model performance was assessed using training and validation datasets. The evaluation process was as follows: (1) the area under the curve (AUC) value as well as sensitivity, specificity, accuracy, and 95% confidence intervals (CI) were evaluated for both training and validation datasets; (2) the receiver operating characteristic (ROC) curve and the AUC value were used to assess the predictive effectiveness of different models in both datasets; (3) the calibration curve was used to assess the nomogram calibration; and (4) the decision curve analysis (DCA) assessed the clinical efficiency of each model.

### 2.7 Statistical analysis

R (version 3.6.2) and SPSS software (version 24.0) were used for the statistical analysis. Continuous variables in the study were expressed as median (interquartile range) or mean ± standard deviation, while categorical variables were expressed as numbers and percentages. The Shapiro-Wilk test was used to evaluate the normality of the distribution. Variables that differed significantly between the training and validation datasets were detected using two-sample t-tests, U-tests, or chi-squared tests. Model construction was completed using LASSO regression or logistic regression. The “rms” and “rmda” packages of R were used to construct calibration and DCA, respectively. Statistical significance was set at P< 0.05.

## 3 Results

### 3.1 Clinicopathological characteristics

The 242 enrolled patients were randomly divided into training (n = 170) and validation (n = 72) datasets. The training dataset consists of 147 men and 23 women. Their ages were 60 (50.75–65.25) years for the high-grade patients and 59 (50.00–66.75) years for the low-grade patients. Correspondingly, 64 men and 8 women comprised the validation dataset; their ages were 56.11 ± 13.14 years for the high-grade patients and 58.24 ± 10.41 years for the low-grade patients. The clinicopathological characteristics of the training dataset did not differ significantly from those of the validation dataset (P > 0.05). The clinicopathological characteristics of the patients are listed in **Table 1**.

**Table 1:** Patients’ characteristics in the training and validation datasets.

Characteristics	Training dataset (n=170)			Test dataset (n=72)			P_inter_
	Low-grade	High-grade	P_intra_	Low-grade	High-grade	P_intra_	
Age	59(50.00~66.75)	60.00(50.75~65.25)	0.926	58.24 ± 10.41	56.11 ± 13.14	0.447	0.720
Gender							
male	83(83.00)	64(91.43)	0.114	32(86.49)	32(91.43)	0.770	0.607
female	17(17.00)	6(8.57)		5(13.51)	3(8.57)		
Smoke							
No	62(62.00)	44(62.86)	0.910	24(64.86)	19(54.29)	0.360	0.701
Yes	38(38.00)	26(37.14)		13(35.14)	16(45.71)		
Drink							
No	71(71.00)	51(72.86)	0.791	24(64.86)	26(74.29)	0.386	0.716
Yes	29(29.00)	19(27.14)		13(35.14)	9(25.71)		
High blood pressure							
No	73(73.00)	56(80.00)	0.294	26(70.27)	26 (74.29)	0.704	0.549
Yes	27(27.00)	14(20.00)		11(29.73)	9(25.71)		
Diabetes							
No	85(85.00)	59(84.29)	0.899	31(83.78)	30(85.71)	0.820	0.997
Yes	15(15.00)	11(15.71)		6(16.22)	5(14.29)		
Liver cirrhosis							
Absent	55(55.00)	41(58.57)	0.644	17(45.95)	20(57.14)	0.342	0.468
Present	45(45.00)	29(41.43)		20(54.05)	15 (42.86)		
HBsAg							
Negative	20(20.00)	4(5.71)	0.008	5(13.51)	6(17.14)	0.669	0.815
Positive	80(80.00)	66(94.29)		32(86.49)	29(82.86)		
AFP							
≤200	64(64.00)	33(47.14)	0.029	25(67.57)	18(51.43)	0.163	0.701
>200	36(36.00)	37(52.86)		12(32.43)	17(48.57)		
TBIL	13.80(10.40~18.68)	16.25(11.65~23.35)	0.233	18.60(11.75~27.00)	15.20(8.50~19.00)	0.049	0.582
DBIL	5.05(3.60~6.95)	5.30(4.25~6.90)	0.352	6.60 (4.00~9.65)	5.10(3.30~6.20)	0.064	0.369
IBIL	8.50(6.40~12.10)	10.15(7.73~13.08)	0.223	12.60(7.05~16.90)	9.30(5.10~12.90)	0.043	0.653
ALT	32.00(21.00~48.00)	40.00(24.00~60.25)	0.085	41.00(21.00~68.50)	28.00(19.00~49.00)	0.029	0.610
AST	33.00(24.00~52.00)	41.50(27.75~58.50)	0.034	38.00(28.50~78.00)	36.00(28.00~42.00)	0.156	0.318
BUN	5.08(4.50~6.00)	4.75(4.02~5.98)	0.193	5.05 (4.36~6.12)	5.17 (4.53~5.76)	0.748	0.426
Scr	66.95(61.48~77.15)	66.25(56.43~75.00)	0.326	70.20(61.00~77.10)	66.00(59.20~73.80)	0.267	0.949
TC	4.28(3.81~4.96)	4.46(3.92~4.89)	0.530	4.43 (3.81~4.95)	4.24(3.87~5.12)	0.673	0.929
TG	0.92(0.74~1.30)	0.92(0.72~1.22)	0.516	0.96 (0.79~1.33)	0.97(0.78~1.59)	0.844	0.185
RBC	4.43(4.19~4.91)	6.60(4.33~4.90)	0.134	4.50 (4.09~4.80)	4.64(4.34~4.91)	0.226	0.878
NEU	3.02(2.49~3.98)	3.84(2.80~5.17)	0.004	2.79 (2.07~4.39)	3.12(2.06~4.17)	0.809	0.143
LYMPH	1.67(1.39~2.16)	1.67(1.27~2.09)	0.266	1.43 ± 0.45	1.84 ± 0.73	0.005	0.099
PLT	180.5(129.25~221.75)	185.00(141.75~231.50)	0.391	182.00(121.00~243.50)	189.00(159.00~244.00)	0.171	0.336
NLR	1.81(1.40~2.32)	2.33(1.76~3.09)	<0.001	1.97 (1.32~3.27)	2.07(1.40~2.82)	0.924	0.860
PLR	99.43(75.70~131.33)	110.2(84.63~150.09)	0.132	118.27(88.26~159.47)	117.68(93.73~147.93)	0.973	0.056
SII	313.5(217.87~497.31)	440.30(273.17~707.15)	0.007	329.12(187.83~621.22)	460.14(254.46~581.32)	0.186	0.779

P_intra_ is the result of univariate analyses between the Low-grade and High-grade groups while P_inter_ represents whether a significant difference exists between the training and validation datasets.

### 3.2 Clinical model construction and evaluation

HBsAg, AST, AFP, NEU, NLR, and SII reached statistical significance in the univariate analysis (P< 0.05). Logistic regression analysis indicated that AFP, NEU, and HBsAg were independent preoperative predictors for HCC pathological grade (**Table 2**) used to construct the clinical model. The clinical model had unsatisfactory AUC values (0.700 for the training dataset and 0.526 for the validation dataset). Therefore, this clinical model is not a suitable tool for pathological grade prediction.

**Table 2:** Multivariate analysis of the preoperative clinical data.

Characteristics	Coefficient	P value	OR	95% CI of OR
HBsAg	1.418	0.018	4.129	(1.279,13.328)
AFP	0.716	0.034	2.047	(1.056,3.969)
AST	-0.002	0.593	0.998	(0.993,1.004)
NEU	0.356	0.018	1.427	(1.063,1.917)
NLR	-0.191	0.175	0.826	(0.627,1.088)
SII	0.001	0.477	1.001	(0.999,1.002)
Constant	-3.007	<0.001	0.049	-

Clinical=-3.007 + 1.418*HBsAg+0.716*AFP (define AFP≦200 as 0, AFP>200 as 1)+0.356*NEU.

### 3.3 Radiomics model construction and evaluation

**Figure 3:**
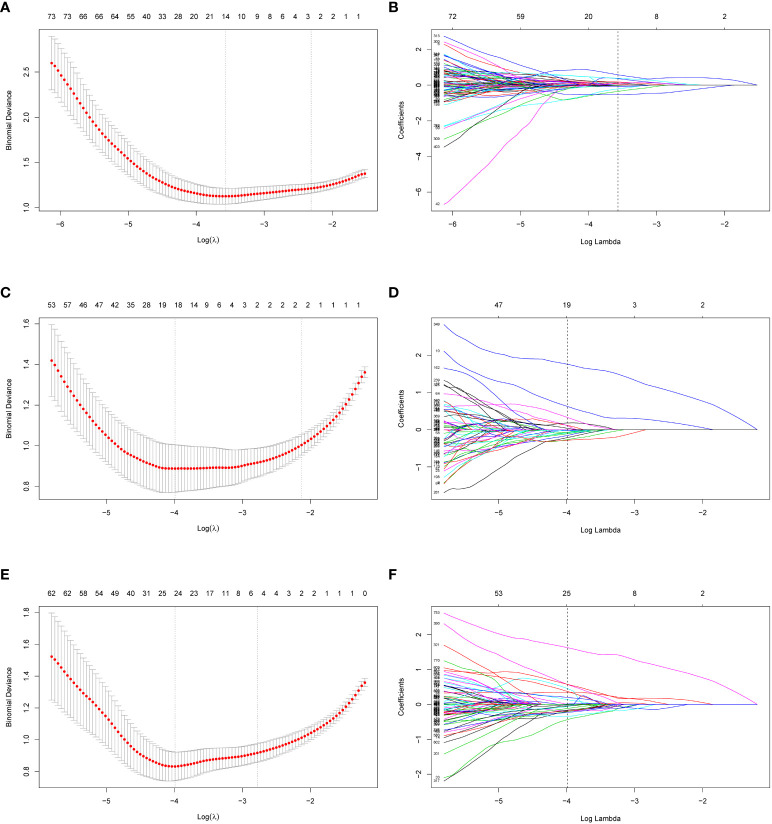
Radiomics feature selection of AP, VP and AP+VP using LASSO regression. Using a minimum criterion, 10-fold cross-validation was employed to select the tuning parameter (lambda) in the LASSO models for AP **(A)**, VP **(C)** and AP+VP **(E)**. Profiles of LASSO coefficients of radiomic features of AP **(B)**, VP **(D)** and AP+VP **(F)**. There is a y-axis for coefficient value, a lower x-axis for log (lambda) and a top x-axis for the number of non-zero coefficients. Based on 10-fold cross-validation, the vertical line was drawn at the optimal value of lambda. AP arterial phase, VP venous phase.

In total, 2632 radiomic features were extracted (1316 from the AP images and 1316 from the VP images). First, after evaluating intra-observer and inter-observer reliability, 951 AP features, 928 VP features, and 1879 combined AP and VP (AP+VP) features with ICC > 0.75 in both intra- and inter-observer reliability, were preliminarily retained. Second, the univariate analysis selected 404 AP, 369 VP, and 773 AP+VP features for further regression analysis. Finally, 14, 19, and 24 features were selected for constructing the AP, VP, and AP+VP radiomics models, respectively, after LASSO regression analysis (**Figure 3**). In both the training and validation datasets, the AP+VP model had a better prediction performance (AUC = 0.981 and 0.842, respectively) than the AP (AUC = 0.890 and 0.773, respectively) and VP (AUC = 0.944 and 0.833, respectively) models. The radiomic features of the 3 radiomics models are listed in **Table 3**.

**Table 3:** Features contained in radiomics models.

Radiomics model	Feature
AP model	AP_log.sigma.2.0.mm.3D_glszm_ZoneEntropy AP_wavelet.LHH_glcm_JointEntropy AP_original_glszm_LowGrayLevelZoneEmphasis AP_original_glszm_SmallAreaLowGrayLevelEmphasis AP_log.sigma.1.0.mm.3D_gldm_DependenceEntropy AP_log.sigma.1.0.mm.3D_gldm_LowGrayLevelEmphasis AP_log.sigma.5.0.mm.3D_glszm_SmallAreaHighGrayLevelEmphasis AP_wavelet.LLH_firstorder_Uniformity AP_wavelet.LLH_glcm_Contrast AP_wavelet.LHL_glszm_LargeAreaHighGrayLevelEmphasis AP_wavelet.HLL_glcm_InverseVariance AP_wavelet.HLL_glszm_LargeAreaHighGrayLevelEmphasis AP_wavelet.LLL_glcm_Idn AP_wavelet.LLL_glcm_MCC
VP model	VP_log.sigma.2.0.mm.3D_glszm_ZoneEntropy VP_original_shape_Maximum2DDiameterSlice VP_wavelet.LLH_glszm_ZoneEntropy VP_original_glrlm_LowGrayLevelRunEmphasis VP_log.sigma.1.0.mm.3D_glszm_ZoneEntropy VP_log.sigma.1.0.mm.3D_gldm_DependenceEntropy VP_log.sigma.1.0.mm.3D_gldm_DependenceNonUniformityNormalized VP_log.sigma.3.0.mm.3D_ngtdm_Contrast VP_log.sigma.5.0.mm.3D_glszm_HighGrayLevelZoneEmphasis VP_log.sigma.5.0.mm.3D_glszm_LargeAreaHighGrayLevelEmphasis VP_wavelet.LLH_glrlm_ShortRunEmphasis VP_wavelet.LHL_glcm_Idn VP_wavelet.LHH_glcm_Autocorrelation VP_wavelet.HLL_glszm_LargeAreaEmphasis VP_wavelet.HLL_glszm_SmallAreaLowGrayLevelEmphasis VP_wavelet.HLH_glszm_GrayLevelNonUniformity VP_wavelet.LLL_firstorder_MeanAbsoluteDeviation VP_wavelet.LLL_glcm_MCC VP_wavelet.LLL_ngtdm_Contrast
AP+VP model	AP_log.sigma.2.0.mm.3D_glszm_ZoneEntropy AP_original_glszm_LowGrayLevelZoneEmphasis AP_original_gldm_LargeDependenceLowGrayLevelEmphasis AP_log.sigma.1.0.mm.3D_glcm_Contrast AP_log.sigma.5.0.mm.3D_glszm_SmallAreaHighGrayLevelEmphasis AP_wavelet.HLL_glszm_LargeAreaLowGrayLevelEmphasis AP_wavelet.HLL_gldm_DependenceEntropy AP_wavelet.HLH_glszm_GrayLevelNonUniformity AP_wavelet.HHH_firstorder_Variance AP_wavelet.LLL_glcm_Idn VP_original_shape_Maximum2DDiameterSlice VP_original_gldm_LowGrayLevelEmphasis VP_log.sigma.1.0.mm.3D_glszm_ZoneEntropy VP_log.sigma.2.0.mm.3D_glszm_ZoneEntropy VP_log.sigma.3.0.mm.3D_glrlm_RunLengthNonUniformityNormalized VP_log.sigma.3.0.mm.3D_ngtdm_Contrast VP_log.sigma.5.0.mm.3D_glszm_HighGrayLevelZoneEmphasis VP_wavelet.LLH_glrlm_ShortRunEmphasis VP_wavelet.LHL_glcm_Idn VP_wavelet.HLL_glszm_SmallAreaLowGrayLevelEmphasis VP_wavelet.HLH_glszm_GrayLevelNonUniformity VP_wavelet.LLL_firstorder_MeanAbsoluteDeviation VP_wavelet.LLL_glcm_MCC VP_wavelet.LLL_ngtdm_Contrast

### 3.4 Combined model construction and evaluation

Using logistic regression, combined models (AP+clinical, VP+clinical, and AP+VP+clinical) were built using the factors from the clinical model and Radscores. In both the training and validation datasets, the AP+VP+clinical model (AUC = 0.985 and 0.829, respectively) performed better than the AP+clinical (AUC = 0.904 and 0.740, respectively) and VP+clinical models (AUC = 0.950 and 0.821, respectively).

### 3.5 Model comparison and nomogram construction

**Figure 4:**
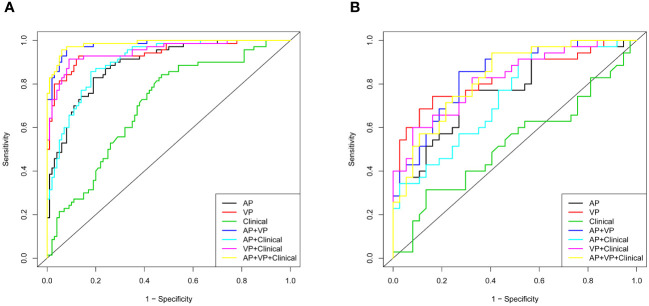
Different models with receiver operating characteristic curves (ROC). **(A)** Training cohort; **(B)** validation cohort.

In **Figure 4**, all seven models are shown along with their ROC curves. The detailed prediction performances of the seven models are presented in **Table 4**. Although a higher AUC value was observed for the AP+VP+clinical model (AUC = 0.985) than for the AP+VP model (AUC = 0.981) in the training dataset, the AP+VP model performed better in the validation dataset (AUC = 0.842).

**Table 4:** Predictive performance of different models.

Models	Training dataset (n=170)				Test dataset (n=72)			
	Sensitivity	Specificity	Accuracy	AUC(95%CI)	Sensitivity	Specificity	Accuracy	AUC(95%CI)
AP	0.829	0.810	0.818	0.890(0.843-0.938)	0.743	0.730	0.736	0.773(0.665-0.881)
VP	0.929	0.870	0.894	0.944(0.908-0.980)	0.743	0.838	0.791	0.833(0.737-0.929)
Clinical	0.843	0.530	0.659	0.700(0.621-0.779)	0.314	0.865	0.597	0.526(0.389-0.663)
AP+VP	0.971	0.920	0.941	0.981(0.965-0.997)	0.857	0.730	0.791	0.842(0.752-0.931)
AP+ Clinical	0.857	0.820	0.835	0.904(0.861-0.948)	0.971	0.432	0.694	0.740(0.626-0.853)
VP+ Clinical	0.914	0.910	0.911	0.950(0.917-0.983)	0.600	0.919	0.763	0.821(0.725-0.917)
AP+VP+Clinical	0.957	0.940	0.947	0.985(0.971-0.998)	0.943	0.595	0.763	0.829(0.737-0.922)

AP, arterial phase; VP, portal venous phase; AUC, area under the curve; CI, confidence interval.

**Figure 5:**
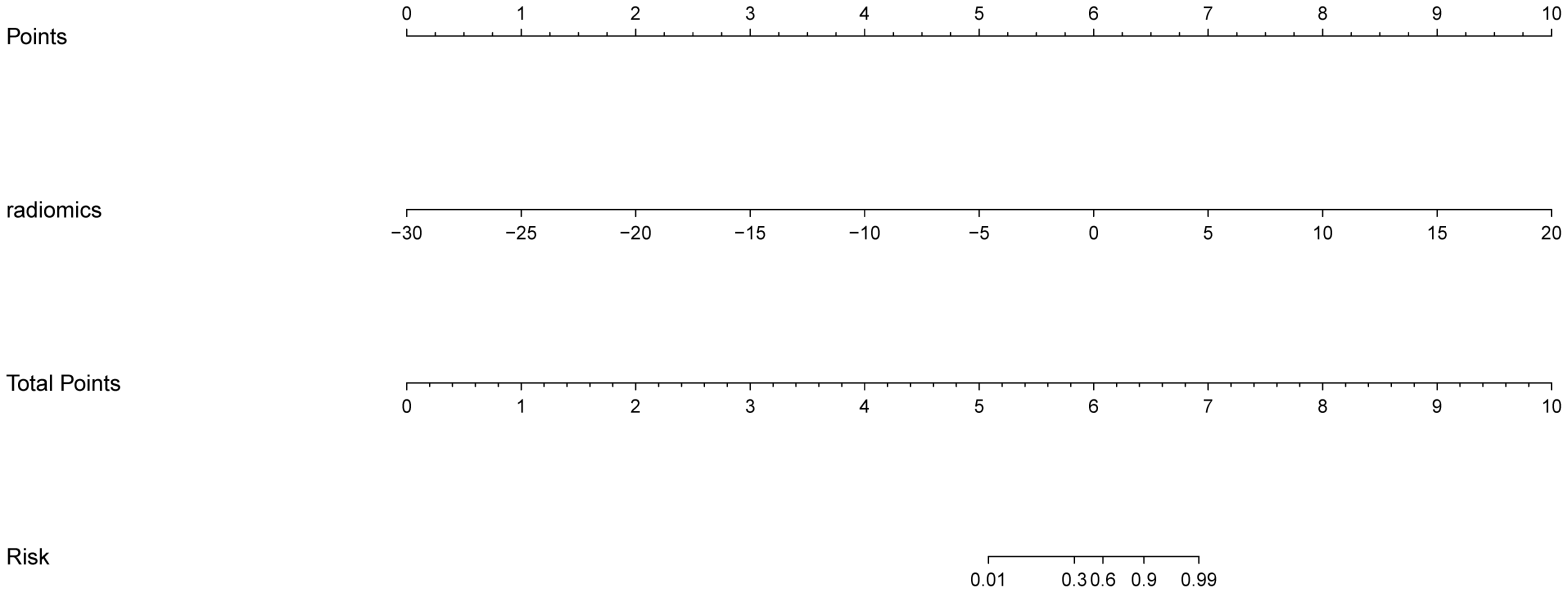
A nomogram for predicting poorly differentiated HCC risk.

**Figure 6:**
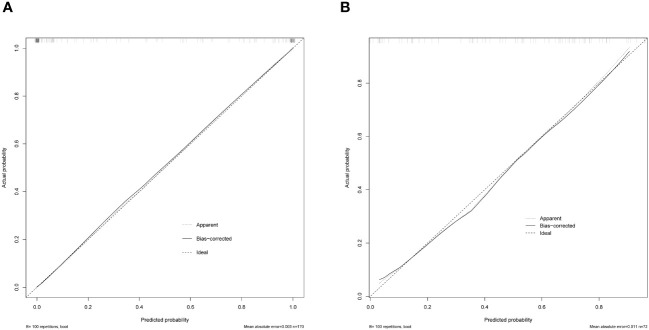
Model calibration curves for the train **(A)** and test cohorts **(B)** for the AP+VP model.

**Figure 7:**
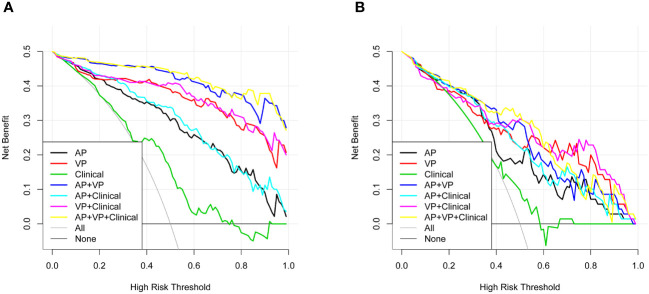
Decision curve analysis for the radiomics nomogram and the clinical factors model in the train dataset **(A)** and validation dataset **(B)**. An x-axis indicates the probability of reaching a threshold; a y-axis indicates the net benefit. As indicated by the threshold probabilities obtained, the radiomics nomogram (blue line) provided a greater net benefit than the clinical factors model (green line).

The AP+VP model was used to establish a nomogram to predict the pathological grade of HCC because of its superior performance during model evaluation (**Figure 5**). The nomogram calibration curves in the training and validation datasets demonstrated good calibration (**Figure 6**). In the Hosmer-Lemeshow test, the nomogram suggested a satisfactory fit because of the non-significant results (P > 0.05). The DCA presented in **Figure 7** shows a higher overall net benefit of the AP+VP model in predicting the HCC pathological grade compared with the clinical model across most of the range of reasonable threshold probabilities.

## 4 Discussion

HCC is a highly heterogeneous cancer with a poor prognosis (23). The heterogeneity and poor prognosis of HCC are associated with tumor pathological grade. For example, low-grade HCCs have lower rates of recurrence and mortality than high-grade HCCs (24), and a high pathological grade is also associated with portal vein invasion in HCCs (25–27), which affects the selection of surgical timing and protocols. Thus, the pathological grade of HCC is important when selecting treatment options. However, definitive diagnosis of the HCC pathological grade is still difficult by non-invasive means. To solve this problem, we constructed a radiomics prediction model for the pathological grade of HCC and successfully confirmed its efficiency.

Previous studies have provided a variety of ways to differentiate between high- and low-grade HCC. Li et al. (17) developed a nomogram that included inflammatory biomarkers for predicting the histological grade of HCC, and the nomogram was confirmed to be reliable, with an AUC value of 0.727. Zhang et al. (28) developed a prediction model (AUC = 0.942) to distinguish patients with HCCs of different pathological grades based on metabolomic biomarkers. Our study identified AFP, NEU, and HBsAg levels as independent predictors of the differential diagnoses of low- and high-grade HCC. Interestingly, we found that neutrophil count is a strong pathological grade predictor of HCC among inflammatory biomarkers, which is consistent with previous studies (18). Peripheral blood NEU count is higher in patients with poorly differentiated HCC; however, the underlying mechanism is difficult to understand. One possible explanation is that neutrophils produce a significant amount of circulating vascular endothelial growth factor, which promotes tumor progression and angiogenesis (29). Previous research has shown that an abnormal HBsAg level may be a signal for poorly differentiated HCCs (18, 30). Wu et al. (31) found a close association between AFP levels and poorly differentiated HCCs. These findings support our results. However, published studies have shown that HCC patients with high- and low-grade disease did not significantly differ in their serological characteristics (21, 32), which might explain why the clinical model performed poorly, with an AUC value of 0.526 in the validation dataset.

Radiomics is a new branch of science that involves extracting quantitative features from images through different mathematical algorithms to improve image analysis and diagnostic performance (33). Our study's ROC curves indicated that the AP+VP model (without laboratory indices) was the best model for pathological grade prediction; therefore, radiomic features can be regarded as more important than other indices. As high-dimensional features, wavelet texture features cannot be easily deciphered by humans but can be used to detect tumor heterogeneity (34, 35), whereas LoG-filtered texture features enhance image grayscale contrast and can also reflect tumor heterogeneity (36, 37). Tumor heterogeneity reflected by wavelet texture features and LoG-filtered texture features explains why 12 wavelet texture features and 8 LoG-filtered texture features played leading roles in pathological grade prediction among the 24 features contained in the AP+VP model.

Two different studies with 101 and 46 patients with HCCs attempted preoperative prediction of the histological grading of HCC using MRI texture features. However, these studies did not consider the predictive value of serum inflammatory biomarkers, and their case numbers were too limited to reserve a validation group (38, 39). Few studies have also aimed at predicting the pathological grade by using CT radiomics. For example, Chen et al. (21) used a support vector machine (SVM) to predict the HCC pathological grade based on CECT radiomics signatures; regrettably, they only considered portal venous phase images and not valuable arterial phase information. Ueda et al. (40) and Matsui et al. (41) suggested that the hepatic artery blood supply increases and the portal vein blood supply decreases when the degree of malignancy of liver tumors increases; in other words, the blood supply can reflect HCC malignancy. The association between blood supply and HCC malignancy may partly explain why both arterial phase and portal venous phase contrast-enhanced CT could predict the HCC pathological grade in our study and why the prediction efficiency was more satisfactory when arterial and portal venous phase images were combined. Furthermore, the multi-phase contrast enhanced MRI may reflect the pathological grading of HCC, with the low-grade lesions exhibiting obvious enhancement, particularly in the portal phase, while the high-grade lesions always displayed enhancement in the arterial phase (42). So, it is easy to appreciate the usefulness of contrast enhancement MRI in discriminating the differentiation grade of HCC. Because of the small number of MRI subjects, our institution has not been able to conduct research related to contrast enhancement MRI and pathological grading of HCC.

Our study has several distinct strengths. First, compared with other studies, our study included many more radiomic features, suggesting that we had a lower likelihood of omitting any crucial radiomic signature. Second, compared with some studies with only 2D ROI (13), our study added more information and improved the reliability of the results by delineating a 3D region including every slice of the tumor. Third, the radiomic analysis in our study involved not only arterial but also portal venous images, while some prior studies used only one type of image (21). Because of the different enhancement patterns of both phases, combined CECT radiomic analysis of arterial and portal venous phase images is regarded as a more useful predictive tool than analysis based only on portal venous images.

Our study has the following limitations: (1) As a retrospective single-center study, the results were limited because of the small sample size. (2) Because of the small number of patients who underwent MRI, we had no opportunity to compare the contrast-enhanced CT and contrast-enhanced MRI radiomics models. (3) The CT images used in our study had a dissatisfactory slice thickness of 5 mm. Reportedly, the diagnostic information might be better obtained from thinner slices (43). (4) There is no clear explanation for the biological mechanisms that produce these imaging features, as in all radiomics studies; therefore, in the future, researchers may be able to gain clear mechanistic insight into the biological basis of these radiomic features by investigating a possible association between radiomics and tumor genomics or proteomics.

## 5 Conclusion

According to our study, the radiomics features from contrast-enhanced CT images could successfully be used to construct a prediction model for categorizing HCC tumors into high- or low-grade cases. However, the combination of the model with clinical data did not perform better than the radiomics model.

## Data availability statement

The raw data supporting the conclusions of this article will be made available by the authors, without undue reservation.

## Ethics statement

The studies involving humans were approved by Ethics Committee of First Affiliated Hospital of Fujian Medical University. The studies were conducted in accordance with the local legislation and institutional requirements. The human samples used in this study were acquired from a by- product of routine care or industry. Written informed consent for participation was not required from the participants or the participants’ legal guardians/next of kin in accordance with the national legislation and institutional requirements.

## Author contributions

YH: Conceptualization, Data curation, Investigation, Formal analysis, Visualization, Writing – original draft. LC: Investigation, Visualization, Writing – original draft. QD: Data curation, Writing – original draft. HZ: Formal analysis, Writing – original draft. YZ: Data curation, Writing – original draft. XZ: Conceptualization, Supervision, Writing – review & editing. SW: Conceptualization, Supervision, Writing – review & editing.
